# Performance Enriching Channel Allocation Algorithm for Vehicle-to-Vehicle City, Highway and Rural Network

**DOI:** 10.3390/s19153283

**Published:** 2019-07-25

**Authors:** Mohammed Abdulhakim Al-Absi, Ahmed Abdulhakim Al-Absi, Hoon Jae Lee

**Affiliations:** 1 Department of Computer Engineering, Graduate School, Dongseo University, 47 Jurye-ro, Sasang-gu, Busan 47011, Korea; 2 Department of Smart Computing, Kyungdong University 46 4-gil, Bongpo, Gosung, Gangwon-do 24764, Korea; 3 Division of Information and Communication Engineering, Dongseo University, 47 Jurye-ro, Sasang-gu, Busan 47011, Korea

**Keywords:** DSRC, ILP, MAC, VANET, V2V, V2I, radio propagation

## Abstract

Future safety applications require the timely delivery of messages between vehicles. The 802.11p has been standardized as the standard Medium Access Control (MAC) protocol for vehicular communication. The 802.11p uses Carrier Sense Multiple Access with Collision Avoidance (CSMA/CA) as MAC. CSMA/CA induces unbounded channel access delay. As a result, it induces high collision. To reduce collision, distributed MAC is required for channel allocation. Many existing approaches have adopted Time Division Multiple Access (TDMA) based MAC design for channel allocation. However, these models are not efficient at utilizing bandwidth. Cognitive radio technique is been adopted by various existing approach for channel allocation in shared channel network to maximize system throughput. However, it induces MAC overhead, and channel allocation on a shared channel network is considered to be an NP-hard problem. This work addresses the above issues. Here we present distributed MAC design PECA (Performance Enriching Channel Allocation) for channel allocation in a shared channel network. The PECA model maximizes the system throughput and reduces the collision, which is experimentally proven. Experiments are conducted to evaluate the performance in terms of throughput, collision and successful packet transmission considering a highly congested vehicular ad-hoc network. Experiments are carried out to show the adaptiveness of proposed MAC design considering different environments such City, Highway and Rural (CHR).

## 1. Introduction

The vehicular Ad-Hoc Network (VANET) has attained wide interest due to the rapid growth of wireless technology. VANET is a special type of Mobile Ad-Hoc Network (MANET), where communication takes place among Vehicle to Vehicle (V2V), Vehicle to Infrastructure (V2I) and the combination of both (V2X). Vehicle-to-vehicle (V2V) communications are considered to be a fundamental part of future smart transport systems [1,2]. As a result, this has led to the growth of various smart transport systems [3]. The user expected to be connected everywhere which makes an immediate requirement in V2V connectivity and many applications are also envisioned. The advantage of such a connected network will aid in improving commuter awareness of traffic conditions, accelerate toll processing, be on the go infotainment access, enable sharing among vehicles, provide better safety and so forth. To support such smart transport systems, the Federal Communications Commission (FCC) of the United States (US) has dedicated 75 MHz of spectrum allocated in the 5.9 GHz band for Dedicated Short-Range Communications (DSRC). Then, the IEEE Wireless Access in Vehicular Environment standard stack (e.g., IEEE 1609.4 and IEEE 802.11p) was designed to cater to communications between vehicles in the DSRC band. As compared with other Ad-Hoc networks, VANET has distinctive characteristics such as dynamically varying topology, the high mobility of vehicle and strict delay constraints. These problems must be considered in designing MAC for VANET to cater for both safe and non-safety application services.

The CSMA/CA (Carrier Sense Multiple Access/Collision Avoidance) based 802.11p [4] has been standardized as the standard MAC protocol for vehicular communication. However, if traffic density is high, it induces high collision probability, specifically for broadcasting packets [5]. Broadcasting plays a significant role in transmitting safety related data such as road condition warnings and vehicle accident warnings. Apart from event driven transmission and Wireless Access in Vehicular Environments (WAVE) [6], Reference [7] also provides an extra layer for WAVE-Basic Service Advertisements (WSAs) and basic safety messages (BSMs) [8]. The WSAs needs to be periodically broadcasted by vehicles to cater non-safety application. The BSMs contain critical information of vehicles such as speed and location. To assure reliable real-time service, each vehicle needs to broadcast and exchange BSMs periodically (i.e., once in every 100 ms) [9].

In the IEEE 802.11p MAC protocol, the vehicle starts transmission directly if the channel sensed is idle. Otherwise, it arbitrarily selects the back-off time from the Contention Window (CW) and initializes the back-off counter. Transmission is initialized when the back-off counter is reached to zero. If more than two vehicles try to access channel simultaneously within two hop distance, a collision occurs and none of the data can be successfully received. Considering this scenario, to resend data the vehicle has to re-compete for channel access. An exponential back-off scheme is presented to decrease the likelihood of contention collision for unicast transmission. However, CSMA/CA has the problem potentially of unbounded channel access delay [10]. If the VANET device has multiple packets, it has to contend for multiple times. Furthermore, 802.11p suffers from interference problems due to hidden terminals, since it cannot use the RTS (Request to Send)/CTS (Clear to Send) mechanisms for broadcasting packets [11]. Considering this scenario too, the collision of the packet cannot even be identified right away. No exponential back-off methods can be used for broadcasting packet sand the likelihood of packet collision is significantly high [5].

To overcome the drawback of IEEE 802.11p, TDMA (time division multiple access) based MAC protocols are presented to provision efficient transmission in VANET [12]. However, their approach is centralized and takes advantage of RSUs (Road side units). As a result, it requires a large amount of RSUs. Hence, it is applicable for City environment. To address this, in Reference [13] presented a distributed MAC to utilize bandwidth efficiently. The model overcomes the bandwidth inefficiency of Space Division Multiple Access (SDMA) [14]. Their model reduces collision in the highway environment. However, their model did not consider maximizing system throughput. Cognitive radio technique has been adopted in Reference [15] to utilize bandwidth efficiently. To maximize system throughput on the multi-channel network, channel sharing technique is been adopted in References [15,16]. Channel allocation on shared channel network is considered to be an NP-hard problem [16]. In Reference [16] presented a theoretical model to solve the NP-hard problem. However, their model did not consider experimental study under different environmental conditions such as city, highway and rural area and besides it induce MAC protocol overhead. Further, number of alternative access technology using 80211ax [17] and 802.11bd [18]. The number of hybrid models is presented along with considering heterogeneous network [19,20,21]. However, none of these models has been evaluated under different radio propagation environments. Additionally, it will incur a high deployment cost and effective coordination is required. Thus, effective MAC scheduling is needed for utilizing resources efficiently irrespective of the network it operates. Further, to overcome the NP-hard problem and MAC protocol overhead for channel allocation in a shared channel network, our paper presents an efficient distributed design for channel allocation that maximizes the system throughput and reduces packet collision.

The research work contribution is as follows:

- Here we formulated the channel allocation problem to maximize system throughput as a linear optimization problem.
- This work presented two algorithms to solve the NP-hard problem for channel allocation in both shared and non-shared channel access.
- We present a Distributed MAC design for shared channel access by integrating MAC overhead into channel allocation.
- The model design brings a trade-off between maximizing system throughput and reducing collision, which is experimentally proven.
- The proposed MAC design is adaptive in nature, for instance, with city, highway and rural, which is experimentally shown.

The organization of this paper is structured as follows. Section 2 describes the extensive research survey work on improving the performance of Vanet so far. The proposed channel allocation model and distributed medium access control (decentralized) are presented in Section 3. The experimental study is carried out in Section 4. The future work and conclusion is described in the last section.

## 2. Related Work

In this section, we describe the state of the art related to MAC protocols used in vehicular ad-hoc networks. In Reference [22], the delay incurred by the 802.11p protocol due to variations in load is studied. The authors in Reference [23] presented a Decentralized Congestion Control (DCC) algorithm for a V2V network in order to support a cooperative smart transport system. The MAC layer uses carrier sensing to detect the busy state of a radio channel before initializing transmission. Their model overcomes the drawback of Enhanced Distributed Channel Access (EDCA) by introducing a queueing technique to handle packet priority for safety related applications. Experiments are conducted to evaluate performance in terms of coverage range and delay reliability under different environment loads for a highway environment. However, DCC adopts a cross-layer design considering a single channel. The authors in Reference [24] presented a cross layer MAC design for VANET. The objective of their design is to minimize interference between a communicating pair at the MAC and routing layer. They also presented a metric to maximize mean Signal to Interference Ratio (SIR) among transmitter and receiver. The experimental outcome shows improvement in terms of packet delivery ratio and throughput. However, their model induces control channel overhead due to computation and the maintenance of the state information of the channel, thus inducing a high risk of collision.

The authors in Reference [25] showed neighborhood knowledge aids in reducing collision. They presented a distributed synchronized beaconing scheduling mechanism, which aids in reducing packet loss in control channel contention for safety applications. However, the network utilization is very low when the traffic is much lower. It assigns slots to the cell with no vehicle, resulting in bandwidth wastage. In Reference [13], they presented a TDMA based MAC named PTMAC (Prediction based TDMA MAC). This aids in addressing packet collision due to hidden node problems under high traffic density. They presented a prediction model for variable traffic density of the two-way network. The outcome shows a reduction in collision for a varied network and traffic density. However, the model did not consider maximizing system throughput.

In order to maximize system throughput, the authors in Reference [15] adopted a cognitive radio technique to design MAC named Enhanced NonCooperative Cognitive division Multiple Access (ENCCMA). They combined FDMA (frequency division multiple access), TDMA and a cognitive radio technique to design a MAC for a shared multi-channel network. They conducted an experimental study of ENCCMA and a comparison with various state-of-arts of MAC. They showed significant performance improvement. However, allocating bandwidth to a vehicle on shared channel is considered to be a NP-hard problem [16]. To address NP-hard problem, Reference [16] presented an approximation algorithm for the cognitive vehicular network. Three constant-factor approximation algorithms are modeled with polynomial time complexity and theoretical performance guarantees. Theoretical guarantees are verified by simulation results. However, their channel allocation is not distributed in nature which induces MAC protocol overhead. In addition,, Reference [17] presented OFDMA based Multiple Access for IEEE 802.11ax. The model aimed to attain better synchronization and overhead reduction with fast back off and better channel sensing. Further, Reference [18] showed the need for reliable, low latency with high throughput for VANET. They presented an alternate radio access technology. They used IEEE 802.11bd instead for DSRC and NR V2X for C-V2X. These models are designed to cope with current dynamic application need and wireless traffic [25]. In Reference [25], presented a dynamic method for adapting rate and power with 1-hop broadcasting packets for each vehicle in multi-application conditions. The model is designed to reduce channel load and meets multiple application scenarios. However, maximizing system utility is not considered. In Reference [26], the authors presented a channel reservation MAC for a multi-channel environment using serial cooperation, using 802.11ax [21]. The model reduced channel access delay and, using cooperative communication, reduced packet collision. However, collision reduction performance was not evaluated by them. A survey conducted by a researcher in Reference [19] showed the need for a novel design considering heterogeneous VANET [20,21]. In Reference [20], for improving scalability and bandwidth utilization, they presented a distributed context-aware heterogeneous VANET association that dynamically selects its radio access technology in an autonomous manner based on user/application QoS prerequisite. In Reference [21], for enhancing quality of experience (QoE), they presented a hybrid model by combining a software defined network with VANET. Using SDN, the available bandwidth from both Wi-Fi and LTE is used for VANET communication. Further, using vehicle states’ information, the resources are allocated to vehicles with radio access technology that maximize the QoE of all vehicles. An extensive research survey was carried out, which showed that a new MAC design is required to maximize system throughput and minimize MAC overhead. The future MAC design should be adaptive in nature, that is, the design should consider different radio propagating environments such as city, highway and rural areas. In the next section, the problem formulation is presented.

## 3. Problem Formulation

This work aims at maximizing the network throughput for performing channel allocation. Let the throughput achieved by vehicle x be $S_{x}$ and $e_{\mathrm{xy}}$ denotes the channel allocation decision. If channel y is allocated to vehicle x, then $e_{\mathrm{xy}}$ is set to 1, otherwise $e_{\mathrm{xy}}$ is set to 0. The throughput gain problem can be expressed as

(1) $$\underset{E}{\max}\overset{R}{\underset{x}{\sum }}S_{x}.$$

where R is the total number of vehicle in vehicular ad-hoc network. Further, we have the following bound for non-shared channel allocation as

(2) $$\overset{R}{\underset{x}{\sum }}e_{\mathrm{xy}}=1\forall y.$$

We can now formulate the throughput attained by vehicle x on non-shared channel allocation as follows. Let $V_{x}$ be the channel set exclusively allocated to vehicle x and $l_{\mathrm{xy}}$ be the likelihood that channel y is accessible to vehicle x. For easiness, we consider that $l_{\mathrm{xy}}$ are not dependent on each other. Therefore, the $S_{x}$ can be computed as follows

(3) $$S_{x}=1-\underset{y\in V_{x}}{\prod }l_{\mathrm{xy}}^{\prime }=1-\overset{T}{\underset{y=1}{\prod }}{\left(l_{\mathrm{xy}}^{\prime }\right)}^{e_{\mathrm{xy}}}$$

where $l_{\mathrm{xy}}^{\prime }=1-l_{\mathrm{xy}}$ is the likelihood that channel y is not accessible for vehicle x and $1-\underset{y\in V_{x}}{\prod }l_{\mathrm{xy}}^{\prime }$ is the likelihood that utmost one channel is accessible to vehicle x. Since each vehicle can utmost uses one accessible channel, its maximum throughput is one. In the shared channel allocation technique, the bounds in Equation (2) are not required. Hence, it can be seen that solving Equations (1) and (2) is a, NP-Hard problem, since it is a nonlinear integer program. In the next section, the PECA algorithm is presented.

## 4. Performance Enriched Channel Allocation (PECA) Algorithm

Here, we present a low-complexity and efficient technique for channel allocation for non-shared and shared channel vehicular networks. This work considers a distributed vehicular ad-hoc network in which R vehicles exploit spectrum opputunities in T channels. We also consider that each vehicle is within its communication radius (i.e., each vehicle can hear transmission of other vehicles). In addition, at most one channel can be accessed by each vehicle for performing data transmission. In addition, vehicles perform sensing on allocated channels at the beginning of each cycle to explore available channels for communication, where time is divided into a fixed-size of cycle. We also consider that perfect sensing can be accomplished with no sensing error.

### 4.1. Non-Shared Channel Allocation Algorithm (NSCA)

Let $V_{x}$ be the set of channel exclusively allocated for vehicle x (i.e., $V_{x}\cap V_{y}=\emptyset$, x≠y). The NSCA technique iteratively assigns a channel to vehicle to maximize the network throughput.

In each iteration of channel assignment, each vehicle x computes its gain in throughput if the best channel is allocated considering the following condition

(4) $$y_{x}^{\prime }=\underset{y\in V_{z}}{\mathrm{argmax}}l_{x,y},$$

The gain in throughput can be computed as

(5) $${\delta S}_{x}=S_{x}^{z}-S_{x}^{q}=\left[1-\left(1-l_{{\mathrm{xy}}_{x}^{\prime }}\right)\underset{y\in V_{x}}{\prod }\left(1-l_{\mathrm{xy}}\right)\right]-\left[1-\underset{y\in V_{x}}{\prod }\left(1-l_{\mathrm{xy}}\right)\right]=l_{{\mathrm{xy}}_{x}^{\prime }}\underset{y\in V_{x}}{\prod }\left(1-l_{\mathrm{xy}}\right)$$

As can be observed from Equation (5), ${\delta S}_{x}$ will be decreased with every iteration of allocation, because as $V_{x}$ increases, $\underset{y\in V_{x}}{\prod }\left(1-l_{\mathrm{xy}}\right)$ tends to zero. By considering this condition, the proposed Non-Shared Channel Allocation Algorithm (NSCA) is defined in Algorithm 1.

**Table:** 

**Algorithm 1:** Non-Shared Channel Allocation Algorithm
Step 1. Input set of accessible channel $V_{z}=\left\{1,2,3,\ldots ,T\right\}$ & $V_{x}=\emptyset$ for x=1,2,3,…,R.
Step 2. For x=1;x<=R;R++
Step 3. $y_{x}^{\prime }=\underset{y\in V_{z}}{\mathrm{argmax}}l_{x,y}$.
Step 4. If ($V_{x}=\emptyset$) then
Step 5. Obtain ${\delta S}_{x}=S_{x}^{z}-S_{x}^{q}$, where $S_{x}^{q}$ and $S_{x}^{z}$ are the throughputs before and after channel allocation $y_{x}^{\prime }$.
Step 6. Else
Step 7. Obtain ${\delta S}_{x}=l_{{\mathrm{xy}}_{x}^{\prime }}$.
Step 8. End If
Step 9. End For
Step 10. $x^{\prime }=\underset{x}{\mathrm{argmax}}{\delta V}_{x}$.
Step 11. Allocate channel ${y^{\prime }}_{x^{\prime }}$ to vehicle $x^{\prime }$.
Step 12. Update $V_{z}=V_{z}/{y^{\prime }}_{x^{\prime }}$.
Step 13. If $V_{z}$ is empty, terminate the process.
Step 14. Else, go to step 2.

Note: After executing Algorithm 1, we can obtain the set of the channel assigned to each vehicle, based on which the throughput can be computed using Equation (3).

### 4.2. Shared Channel Allocation Algorithm (SCA)

Channel sharing aids in improving the throughput performance of the multi-user vehicular Ad-hoc network. However, the MAC protocol incurs overhead due to channel access contention under multi-user channel assignment. Therefore, a refined channel allocation technique is required to balance throughput gain and Design overhead.

Our channel allocation model is composed of two stages. Firstly, we compute the channel assignment information of a single vehicle using Algorithm 1. Then, we process a multi-user channel assignment by allocating the channels that have been allocated to some vehicles, to other vehicles in the second stage. Now we are ready to model an SCA Algorithm 2 similar to NSCA Algorithm 1. However, computing the metric is quite a challenging and difficult task. Therefore, we compute throughput gain for channel allocation considering MAC overhead D<1. Note that the Overhead D depends on the resultant of channel allocation (i.e., number of channels allocated to different vehicle). The computation of D is shown in a later subsection of this paper.

Let us consider a setup where channel y is the shared channel of vehicle $x_{1},x_{2},x_{3},\ldots ,x_{V}$, where V is the number of vehicles which share the channel. Here we compute throughput gain for a specific vehicle x if channel y is allocated to this vehicle. In fact, this throughput gain can be accomplished, because vehicle x may utilize channel y if this channel is not utilized by other vehicles $x_{1},x_{2},x_{3},\ldots ,x_{V}$ or the channel is not accessible. The throughput gain of vehicle x and channel y considering practical scenario (i.e., $l_{x,y}$ is closer to 1 (e.g., at least 0.8) is computed as

(6) $${\delta S}_{x}^{V,b}\left(y\right)=\left(1-\frac{1}{V}\right)\left(1-D\right)l_{\mathrm{xy}}\left(\prod_{o\in V_{x}}{\bar{l}}_{\mathrm{xo}}\right)*\left(1-\prod_{o\in V_{*}^{C}}{\bar{l}}_{\mathrm{xo}}\right)\sum_{n=1}^{V}\left[{\bar{l}}_{x_{n}y}\left(\prod_{m=1,m\neq n}^{V}l_{x_{m}y}\right)\right].$$

**Algorithm 2:** Shared channel AllocationStep 1. Input set of assigned channels ∀ vehicles $V_{x}=\emptyset$ for x=1,2,3,…,R and $D_{o}$Step 2. Execute Algorithm 1 to get channel allocated for a single vehicle.Step 3. Let the set of channels that are shared by j vehicles be $P_{j}$ and $F_{y}$ be the group of vehicles which share channel y and set $F_{y}^{T}=F_{y}\;\forall y=1,2,3,\ldots ,T$.Step 4. Process=1; o=1; UpdateOvd=0.Step 5. While Process=1 doStep 6. Obtain the set of channels $P_{o}$ shared by o vehicles Step 7.  For $y=1;y\leq \left|P_{o}\right|;y++$Step 8.   For j=1;j≤R;J++Step 9.    If $j\in F_{y}$ thenStep 10.     ${\delta S}_{j}^{o,b}\left(y\right)=0$.Step 11.   ElseStep 12.     User j computes ${\delta S}_{j}^{o,b}\left(y\right)$ considering that channel y is assigned to vehicle j.Step 13.   End IfStep 14.   End ForStep 15.  $j_{y}^{\prime }=\arg\underset{j}{\max}{\delta S}_{j}^{o,b}\left(y\right)$.Step 16.  End ForStep 17. $j_{y}^{\prime }=\arg\underset{y}{\max}{\delta S}_{j_{y}^{\prime }}^{o,b}\left(y\right)$.Step 18. If ${\delta S}_{j^{\prime }}^{o,b}\left({y^{\prime }}_{j^{\prime }}\right)\leq \epsilon$ and UpdateOvd=1 then Step 19.  Set Process=0.Step 20.  Go to step 35.Step 21. End IfStep 22. If ${\delta S}_{j^{\prime }}^{o,b}\left({y^{\prime }}_{j^{\prime }}\right)>\epsilon$ thenStep 23.  Provisionally allocate channel ${y^{\prime }}_{j^{\prime }}$ to vehicle $j^{\prime }$, i.e., update $F_{{y^{\prime }}_{j^{\prime }}}^{T}=F_{{y^{\prime }}_{j^{\prime }}}\cup \left\{j^{\prime }\right\}$.Step 24.  Compute A and D with $F_{{y^{\prime }}_{j^{\prime }}}^{T}$ using Equations (11) and (12), respectively.Step 25.  If $\left|D-D_{o}\right|>\epsilon_{D}$ thenStep 26.  Set:Process=1.Step 27.   Return to Step 7 using the updated $D_{o}=D$.Step 28.  ElseStep 29.   Update $F_{{y^{\prime }}_{j^{\prime }}}=F_{{y^{\prime }}_{j^{\prime }}}^{T}$ (i.e., allocate channel ${y^{\prime }}_{j^{\prime }}$ to vehicle $j^{\prime }$), compute A & $D_{o}$ with $F_{{y^{\prime }}_{j^{\prime }}}$, & update $P_{o}$.Step 30.   Update UpdateOvd=0.Step 31.  End If.Step 32. End If.Step 33. Return to step 7.Step 34. o=o+1.Step 35. End While

### 4.3. Contention Window Computation

To minimize the collision likelihood among contenting vehicle V, the contention window A is computed. In fact, there is a tradeoff among overhead of MAC protocol and collision likelihood, which is influenced by A, that is, a lesser value of A increases the collision likelihood at the cost of lower MAC overhead and vice versa, since each vehicle arbitrarily selects the same back-off time. As a result, the likelihood of the first collision is higher as the number of participating vehicles decreases for each probable collision.

Let ${ℒ}_{u}$ be the likelihood of the first collision. Let us consider the constraint ${ℒ}_{u}\leq \epsilon L$, where ϵL manages the overhead and collision likelihood tradeoff to determine contention window A. Let’s evaluate ${ℒ}_{u}$ as a function of A considering that there are r vehicles in the contention phase. With no loss of generality, let us consider that the arbitrary back-off time of r vehicles is ordered as $g_{1}\leq g_{2}\leq g_{3}\leq \ldots \leq g_{r}$. Assume that, if there are r vehicles in contention phase, the condition likelihood of the first collision can be expressed as

(7) ℒu(r)=∑y=2rL(y vehicles collide)=∑y=2r∑x=0A−2Ury(1A)y(A−x−1A)r−y

where each term in double summation depicts the likelihood that y vehicles collide when they select identical back-off value with respect to x. Therefore, the likelihood of the first collision can be computed as

(8) $${ℒ}_{u}=\overset{R}{\underset{r=2}{\sum }}{ℒ}_{u}^{\left(r\right)}*L\left\{r\;\mathrm{vehicle}\;\mathrm{contend}\right\}$$

where L{r vehicle contend} is the likelihood that r vehicles participate in the contention phase, ${ℒ}_{u}^{\left(r\right)}$ is computed using Equation (7). To evaluate ${ℒ}_{u}$, we derive L{r vehicle contend}. It can be proved that vehicle x participates in contention if utmost one channel in $V_{x}^{C}$ is accessible and all channels in $V_{x}$ are busy. The likelihood of this scenario can be expressed as

(9) $${ℒ}_{C}^{\left(x\right)}=L\left\{\begin{array}{c} there\;exist\;exactly\;one\;channels\;in\;V_{x}^{C}\;\mathrm{are}\;\mathrm{accessible}\;\mathrm{and}\\ \mathrm{all}\;\mathrm{achannel}\;\mathrm{in}\;V_{x}\;\mathrm{are}\;\mathrm{busy} \end{array}\right\}\;=\left(\underset{y\epsilon V_{x}}{\prod }{\bar{l}}_{\mathrm{xy}}\right)\left(1-\underset{y\epsilon V_{x}^{C}}{\prod }{\bar{l}}_{\mathrm{xy}}\right).$$

The likelihood of the scenario that r vehicles participate in contention phase is

(10) $$L\left\{r\;\mathrm{vehicle}\;\mathrm{contend}\right\}=\overset{U_{R}^{r}}{\underset{t=1}{\sum }}\underset{x\in \wedge_{t}}{\prod }{ℒ}_{C}^{\left(x\right)}\underset{x\in \wedge_{R}\backslash \wedge_{t}}{\prod }{ℒ}_{C}^{\left(x\right)}$$

where $\wedge_{R}$ is the set of all R vehicles ({1,2,3,…,R}) and $\wedge_{t}$ is one specific set of r users. Substituting the output in Equation (10) into Equation (8), the ${ℒ}_{u}$ can be computed. Therefore, we can now determine A as

(11) $$A=\min\left\{A|{ℒ}_{u}\left(A\right)\leq \epsilon L\right\}$$

where for simplicity, ${ℒ}_{u}\left(A\right)$ in Equation (8) is denotes as function of A.

### 4.4. MAC Overhead Computation

Using Equation (11), the mean overhead of the MAC protocol can be modeled. Let h be the mean value of a back-off parameter selected by any vehicle. Hence, we have h=(A−1)2 since the back-off value is uniformly selected between zero and A−1 interval (i.e., [0,A−1]). Therefore, the mean overhead can be computed as

(12) $$D\left(A\right)=(\left(\left[A-1\right]\varphi /2)+s_{\mathrm{CTS}}+s_{\mathrm{RTS}}+3s_{\mathrm{SIFS}}+s_{\mathrm{SYNC}}+s_{\mathrm{SEN}}\right)/S_{ℐ}$$

where $s_{\mathrm{CTS}}$, $s_{\mathrm{RTS}}$ and $s_{\mathrm{SIFS}}$ are the corresponding time of CTS (Clear to Send), RTS (Request to Send) and SIFS (Short inter frame space) packets, $s_{\mathrm{SYNC}}$ is the size of synchronization packets, $s_{\mathrm{SEN}}$ is the time of sensing, φ is the time that corresponds to one back-off parameter and $S_{ℐ}$ is the cycle time. The overhead D depends on channel allocation outcome. Therefore, D is updated in Algorithm 2 based on current channel allocation. Our shared channel allocation algorithm is efficient and run smoothly, since D does not change much in two consecutive assignment decisions which are experimentally proved in the next section.

## 5. Simulation Analysis and Result

The experiments were conducted on the Windows 10 operating system, 64-bit I-5 quad core processor with 16 GB RAM and Dedicated 4 GB Nvidia CUDA GPU card. The SIMITS simulator tool was used for experiment evaluation. The Proposed PECA and existing ENCCMA [13] algorithm was written in C# object oriented programing language using Visual studio framework 4.5, 2012. The PECA and city, highway and rural (CHR) radio propagating environment model [Ours] was incorporated into the SIMITS tool [27,28,29,30]. Experiments were conducted to evaluate the performance of PECA over ENCCMA in terms of throughput achieved, successful packet transmission and packet collision. The experiments were conducted considering different environments such as City, Highway and Rural. For all the simulation cases considered in this work, the speed of the vehicle was fixed to 20 cycles per frame, vehicles were varied from 20, 40 and 80 and performance was computed for city, highway and rural environments for both PECA and ENCCMA. Moreover, for evaluating PECA and ENCCMA under dense environments, the following simulation step was considered. Firstly, we considered a minimum of 20 and a maximum of 80 vehicles. Secondly, each vehicle initialized at the same location and they started contending for performing transmission. Then each vehicle started moving from its respective position one after the other. Further, a maximum of 40 slots were available at any given time for contending and performing transmission. Each message requires at least 4 slots for completing the transmission. Thus, a maximum of 10 vehicles can contend and perform transmission at any given time. Under this circumstance, the remaining 70 vehicles (i.e., considering 80 vehicles) will be waiting for contention. Under this setup, an improper scheduling method will lead to a high number of collisions and throughput degradation. Thus, this manuscript evaluates the proposed and existing model under such setups. The simulation parameter considered for evaluation are shown in Table 1.

### 5.1. Throughput Performance

**Figure 1:**
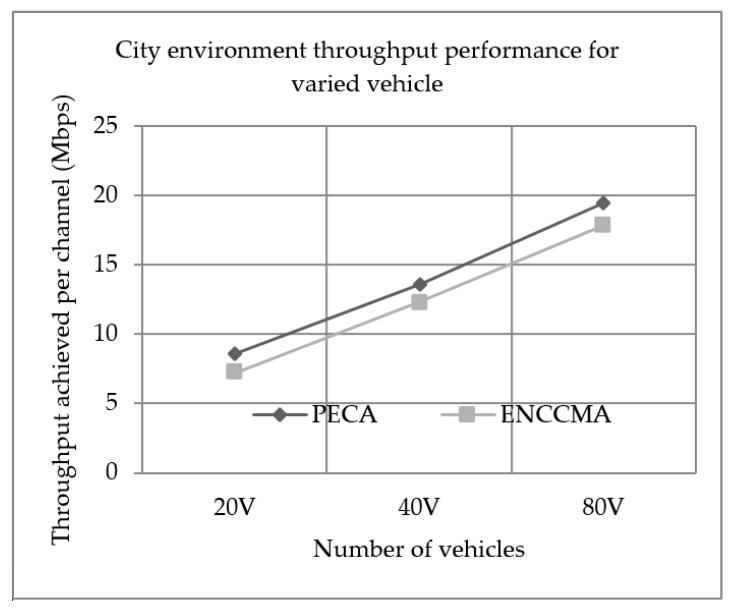
City environment throughput performance for varied vehicle.

**Figure 2:**
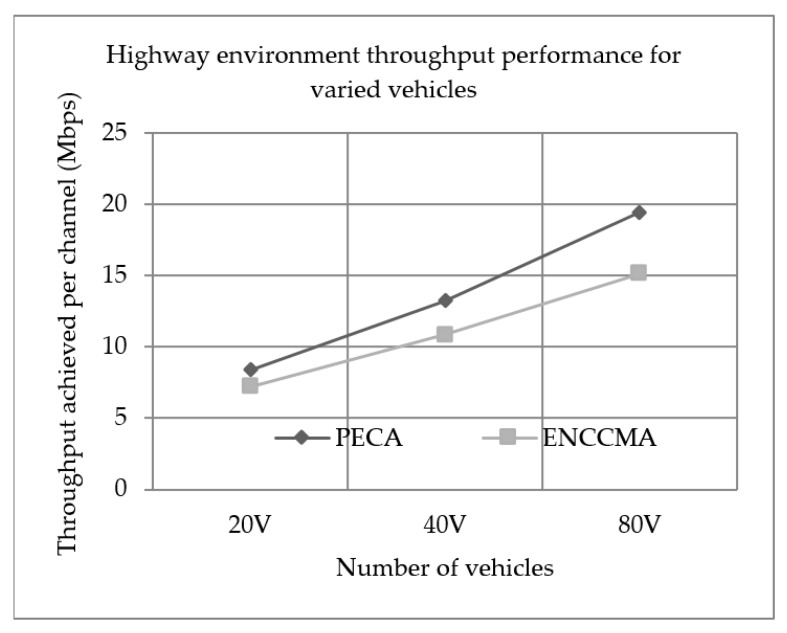
Highway environment throughput performance for varied vehicles.

**Figure 3:**
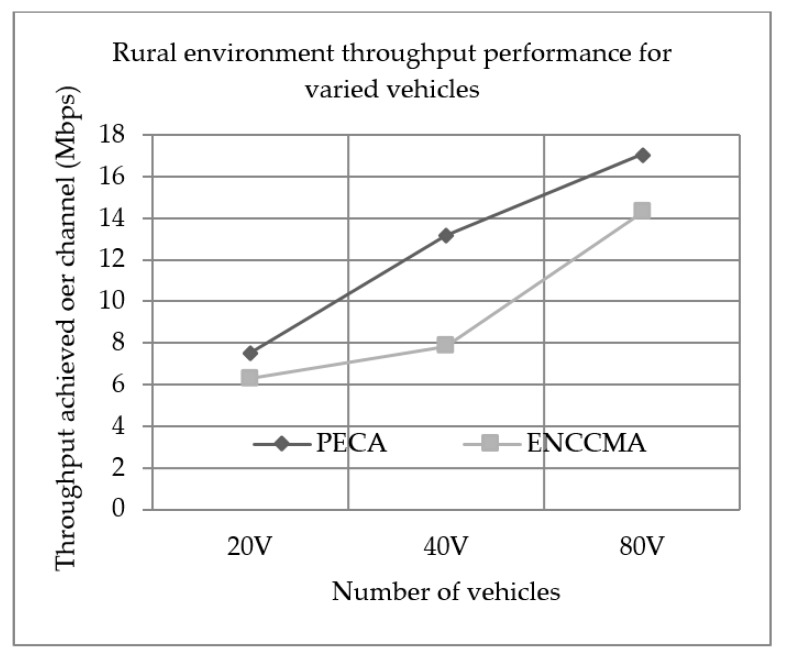
Rural environment throughput performance for varied vehicles.

Experiments were conducted to evaluate the performance of PECA and ENCCMA in terms of throughput achieved per channel for City, Highway and Rural as shown in Figure 1, Figure 2 and Figure 3, respectively, considering different vehicle densities. It is seen from Figure 1, the PECA improves throughput by 16.17%, 9.25% and 8.26% considering 20, 40 and 80 vehicles respectively over ENCCMA. An average throughput improvement of 11.23% was achieved by PECA over ENCCMA for city environment. As seen from Figure 2, the PECA improves throughput by 14.12%, 18.01% and 21.93% considering 20, 40 and 80 vehicles, respectively, over ENCCMA. An average throughput improvement of 18.026% is achieved by PECA over ENCCMA for highway environment. Figure 3 shows the throughput performance for a rural environment. As seen from Figure 3, the PECA improves throughput by 16.42%, 40.38% and 16.17% considering 20, 40 and 80 vehicles respectively over ENCCMA. An average throughput improvement of 24.32% is achieved by PECA over ENCCMA for a rural environment. The overall result achieved shows that PECA performs significantly better than ENCCMA considering varied vehicles and different environmental conditions, which shows the proposed model is adaptive in nature. However, the significant outcome attained by proposed PECA over existing ENCCMA is due to an efficient collision detection method under a shared channel environment. Further, based on collision likelihood information the slots are selected that maximize the system throughput.

### 5.2. Successful Packet Transmission Performance

**Figure 4:**
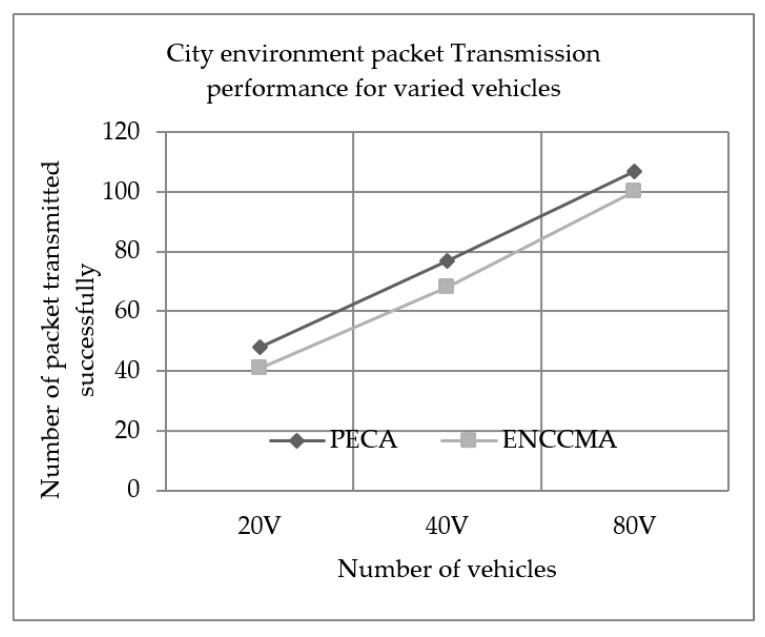
City environment packet Transmission performance for varied vehicles.

**Figure 5:**
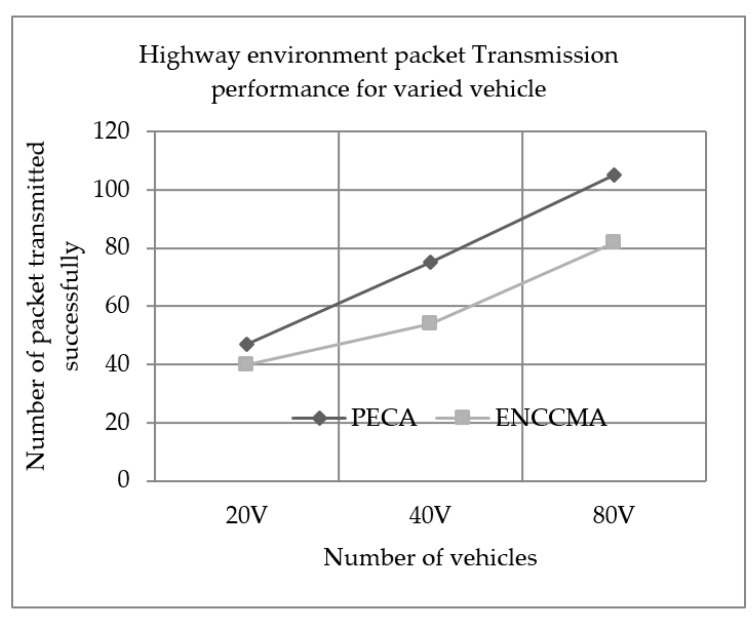
Highway environment packet Transmission performance for varied vehicle.

**Figure 6:**
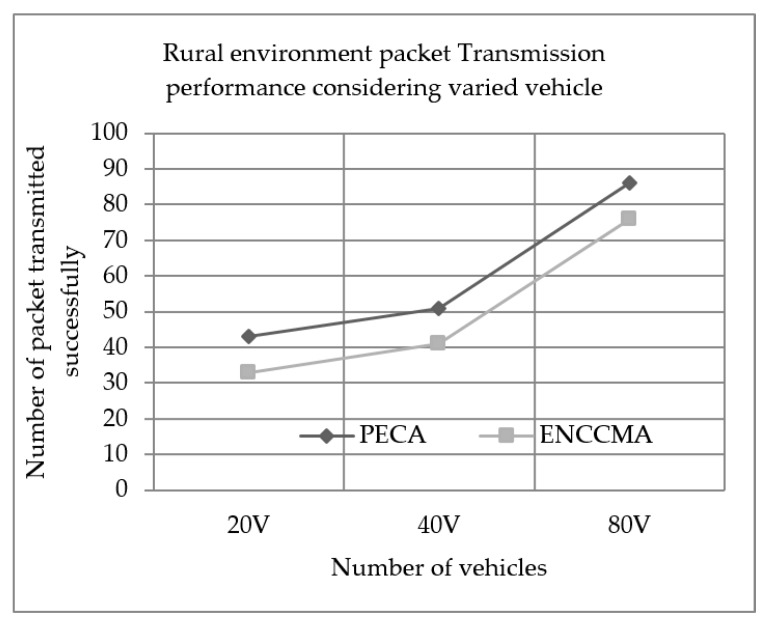
Rural environment packet Transmission performance considering varied vehicle.

Experiments evaluated the performance of PECA and ENCCMA in terms of successful packet transmission for City, Highway and Rural, as shown in Figure 4, Figure 5 and Figure 6, respectively, considering different vehicle densities. As seen from Figure 4, the PECA improves packet transmission by 14.58%, 11.06% and 6.54% considering 20, 40 and 80 vehicles respectively over ENCCMA. An average packet transmission improvement of 10.93% is achieved by PECA over ENCCMA for a city environment. As seen from Figure 5, the PECA improves packet transmission by 14.89%, 28.00% and 21.90% considering 20, 40 and 80 vehicles respectively over ENCCMA. An average packet transmission improvement of 21.59% is achieved by PECA over ENCCMA for a highway environment. The Figure 6 shows the packet transmission performance for a rural environment. It is seen from Figure 6, the PECA improves packet transmission by 23.25%, 19.60% and 11.62% considering 20, 40 and 80 vehicles respectively over ENCCMA. An average packet transmission improvement of 18.16% is achieved by PECA over ENCCMA for a rural environment. The overall result achieved shows that PECA performs significantly better than ENCCMA considering varied vehicles and different environmental conditions in terms of packet transmission. Therefore, the significant outcome attained by the proposed PECA over existing ENCCMA is due to the efficient collision detection method. Thus, it aided in minimizing collision and maximize system throughput. As a result, the packet transmission in the network improved.

### 5.3. Collision Performance

**Figure 7:**
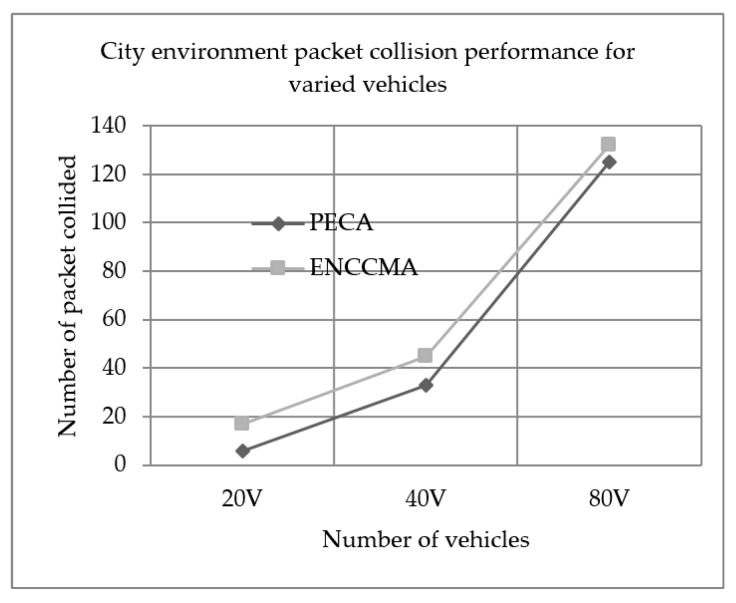
City environment packet collision performance for varied vehicles.

**Figure 8:**
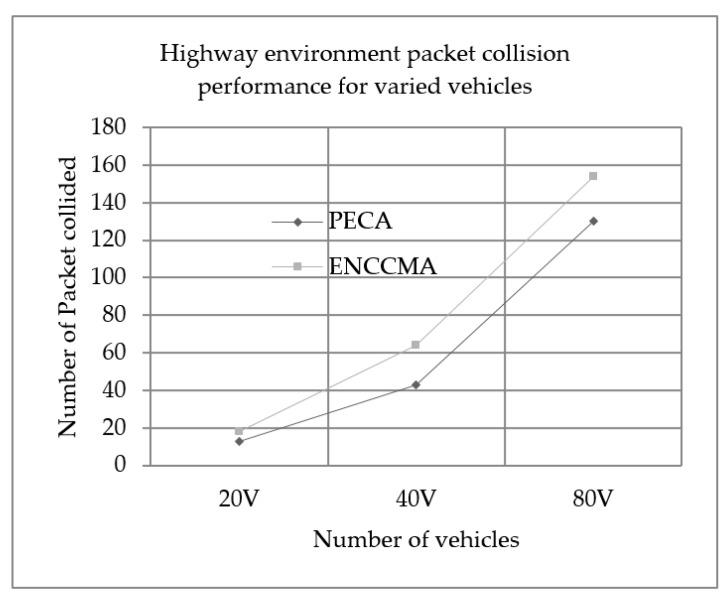
Highway environment packet collision performance for varied vehicles.

**Figure 9:**
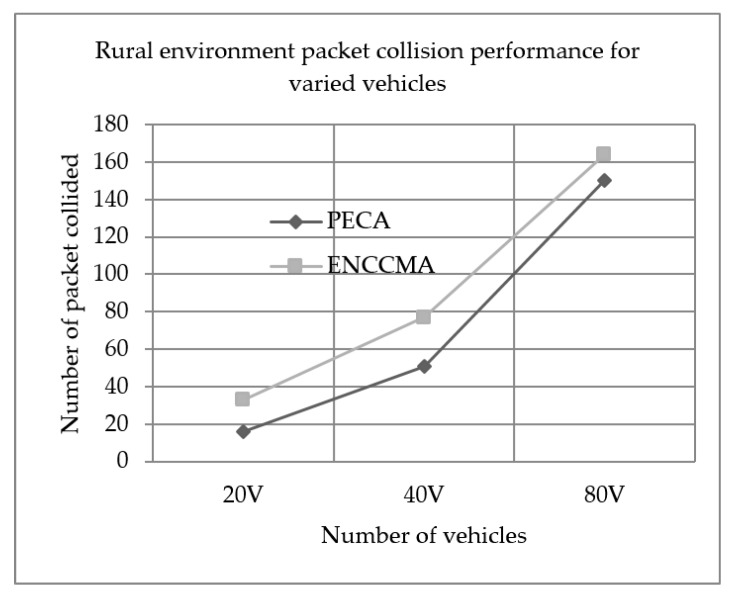
Rural environment packet collision performance for varied vehicles.

We evaluated the performance of PECA and ENCCMA in terms of packet collision for City, Highway and Rural as shown in Figure 7, Figure 8 and Figure 9, respectively, considering different vehicle densities. As seen from Figure 7, the PECA reduces packet collision by 64.70%, 26.66% and 5.3% considering 20, 40 and 80 vehicles respectively over ENCCMA. An average packet collision reduction of 32.22% is achieved by PECA over ENCCMA for a city environment. As seen from Figure 8, the PECA reduces packet collision by 27.77%, 32.81% and 15.58% considering 20, 40 and 80 vehicles respectively over ENCCMA. An average packet collision reduction of 25.39% is achieved by PECA over ENCCMA for a highway environment. Figure 9 shows the packet collision performance for a rural environment. As seen from Figure 9, the PECA reduces packet collision by 51.51%, 33.76% and 08.53% considering 20, 40 and 80 vehicles respectively over ENCCMA. An average packet collision reduction of 31.27% is achieved by PECA over ENCCMA for rural environment. The overall result achieved shows that PECA performs significantly better than ENCCMA considering varied vehicles and different environmental conditions in terms of packet collision. However, the significant outcome attained by the proposed PECA over the existing ENCCMA is due to the efficient back off method post identifying collision in the network under a shared channel environment. Then, the average overhead is computed and updated in the channel allocation process under the shared channel environment. Thus, it aided in minimizing collision in the network.

## 6. Conclusions

This work describes a performance enriched and distribute MAC and for V2V and V2I, namely PECA. PECA overcomes the collision problem due to the channel access delay of CSMA/CA MAC of 802.11p. Here, we showed that the channel allocation problem is an NP-hard non-linear integer programming problem. When each user is given a dedicated channel for transmission for a certain period of time, it is called a non-shared channel allocation. When channels are shared among neighboring vehicles, each vehicle is given a certain amount of time for transmission. Note: in our work, the amount of time required for channel access is defined by two factors, that is, maximizing throughput and minimizing overhead. This work presents an efficient spectrum access mechanism (algorithm) on a multi-channel shared medium access network. The model aims to bring a trade-off between throughput and MAC performance. The model aims to maximize system throughput and minimize MAC overhead (collision). Two algorithms are presented, one for a non-shared channel and then for the shared channel network. First, based on the throughput gain requirement, the user selects the best available channel. Here, the user does not share the channel. The users access the channel for a stipulated session of time and then leave the channel for another user to access it. However, this algorithm does not utilize the bandwidth efficiently since the channel is not shared. To address this, in the second algorithm, a shared channel allocation algorithm is presented. Here, a set of users share the channel among neighboring users. A likelihood estimation for maximizing throughput and minimizing MAC overhead is modeled considering a multi user shared channel network. This algorithm utilizes bandwidth efficiently, which aids in minimizing collision and maximizing system throughput. Experiments are carried out to evaluate the performance of the proposed PECA and the existing ENCCMA in terms of throughput, successful packet transmission and collision. Experiments are carried out considering varied vehicles. The outcomes show significant performance improvement of PECA over ENCCMA, an average throughput improvement of 11.23%, 18.026% and 24.32% achieved by PECA over ENCCMA for city, highway and rural environments respectively, considering varied vehicles, an average packet transmission improvement of 10.93%, 21.59% and 18.16% achieved by PECA over ENCCMA for city, highway and rural environments respectively, considering varied vehicles, an average packet collision reduction of 32.22%, 25.39% and 32.27% achieved by PECA over ENCCMA for city, highway and rural environments respectively, considering varied vehicles. The significant throughput, successful packet transmission and collision outcome are attained due to the development of an efficient collision detection method. Further, using collision likelihood information, the slots are selected that maximize the system throughput. Additionally, the back off information is updated in the channel allocation process, aiding in reducing packet collision and improving successful packet transmission in high density/congested VANET. The overall result achieved shows the adaptiveness of the proposed MAC, considering varied devices and a radio propagating environment. Future work would consider provisioning security among V2V and V2I communication for both ENCCMA and PECA and evaluate their performance.

## Figures and Tables

**Table 1:** Parameters.

Parameters	Value
Network	30 km × 30 km
MAC	ENCCMA & PECA
Modulation scheme	64-QAM
Mobility of Vehicles	20 cycle per frame
Bandwidth	27 Mbps
Frequency Channels	7
Vehicles	40, 20, & 80
Coding rate	0.75
Message size	75 bytes
Time slots	8 μs
Environment	Rural, City & Highway
